# Anatomical factors associated with the development of anterior tibial spine fractures based on MRI measurements

**DOI:** 10.1186/s13018-023-03836-z

**Published:** 2023-05-12

**Authors:** Lei Zhang, Qinghong Xia, Runze Yang, Lei Fan, Yunan Hu, Weili Fu

**Affiliations:** 1 Department of Orthopedics, Orthopedic Research Institute, West China Hospital, Sichuan University, Chengdu, China; 2 Operating Room of Anesthesia Surgery Center, West China Hospital, Sichuan University, Chengdu, China; 3 West China School of Nursing, Sichuan University, Chengdu, China

**Keywords:** Tibial spine fracture, Tibial eminence fracture, Tibial avulsion, Anterior cruciate ligament, Risk factor

## Abstract

**Background:**

Numerous studies have investigated anatomic factors for anterior cruciate ligament (ACL) injuries, such as posterior tibial slope (PTS) and notch width index (NWI). However, anterior tibial spine fracture (ATSF) as a specific pattern of ACL injury, a bony avulsion of the ACL from its insertion on the intercondylar spine of the tibia, has rarely been explored for its anatomical risk factors. Identifying anatomic parameters of the knee associated with ATSF is important for understanding injury mechanisms and prevention.

**Methods:**

Patients who underwent surgery for ATSF between January 2010 and December 2021 were retrospectively reviewed, and 38 patients were included in the study group. Thirty-eight patients who suffered from isolated meniscal tear without other pathologic findings were matched in a 1:1 fashion by age, sex and BMI to the study group. The lateral posterior tibial slope (LPTS), medial posterior tibial slope (MPTS), medial tibial depth, lateral tibial height, lateral femoral condyle ratio (LFCR) and NWI were measured and compared between the ATSF and control groups. Binary logistic regressions identified independent predictors of ATSF. Receiver operator characteristic (ROC) curves were performed to compare the diagnostic performance and determine the cutoff values of associated parameters.

**Results:**

The LPTS, LFCR and MPTS were significantly larger in the knees in the ATSF group than in the control group (*P* = 0.001, *P* = 0.012 and *P* = 0.005, respectively). The NWI was significantly smaller in the knees in the ATSF group than in the control group (*P* = 0.005). According to the results of logistic regression analysis, the LPTS, LFCR and NWI were independently associated with ATSF. The LPTS was the strongest predictor variable, and the ROC analysis revealed 63.2% sensitivity and 76.3% specificity (area under the curve, 0.731; 95% CI 0.619–0.844) for values above 6.9.

**Conclusion:**

The LPTS, LFCR and NWI were found to be associated with the ATSF; in particular, LPTS could provide the most accurate predictive performance. The findings of this study may aid clinicians in identifying people at risk for ATSF and taking individualized preventive measures. However, further investigation regarding the pattern and biomechanical mechanisms of this injury is required.

## Introduction

Anterior tibial spine fractures (ATSFs) or tibial eminence fractures are avulsion fractures of the anterior cruciate ligament (ACL) from its insertion on the tibial intercondylar eminence. ATSFs are rare, with an incidence between 3.0 and 3.5 per 100,000 individuals in the general population [1, 2]. These fractures commonly occur in children and adolescents, predominantly between the ages of 8–14 [3–5]. However, the recent literature suggested that the prevalence of ATSFs in adults is higher than previously reported [6, 7]. The etiology of fractures is various; for pediatric patients, it may be due in part to skeletal immaturity, weak knee muscle tissue and increased ligament elasticity, while for adults, it is mostly due to traffic accidents. The most typical mechanism of injury is knee hyperextension with a valgus or rotational force [8], often resulting from a fall from a bicycle. But these fractures are increasingly common in noncontact injuries in sports, such as skiing and soccer [9].

According to the Meyers and McKeever classification system [10], ATSFs can be classified into 4 types. Type I injury is a minimally displaced fragment. Type II injury involves superior displacement of the anterior bony fragment, while type III and IV injuries involve complete separation of the fragment from the tibia. Type IV injury includes comminution of a displaced avulsion fracture. The different treatment options for ATSFs are full of hot debate, but there is a paucity of literature on the anatomical risk factors for these fractures.

Samora et al. [11] included 25 pediatric patients to evaluate risk factors for ATSF and found no significant differences in posterior tibial slope (PTS) and notch width index (NWI) compared to controls. A previous study by Messner et al. [12] explored the relationship between posterior tibial slope (PTS) and pediatric ATSFs. They found that only male patients undergoing surgical fixation of ATSF had an increased PTS compared with controls. However, their study population was pediatric patients, and their measurement of PTS was performed by plain radiographs, limiting their ability to distinguish the subtle differences between the medial and lateral compartments. In addition to PTS, we want to further explore morphological risk factors for ATSFs of the tibial plateau and femoral condyle. Various osseous morphologic risk factors associated with ACL injuries have been identified in the literature, such as shallower medial tibial depth [13], increased lateral tibial plateau slope [14, 15] and intercondylar notch stenosis [16]. Therefore, the purpose of this study was to determine which anatomic parameters are independently associated with ATSF in adults (1) and the diagnostic values of the individual anatomic parameters (2). It was hypothesized that patients with ATSF would exhibit elevated PTS compared to uninjured controls on MRI measurements.

## Methods

### Patients and study design

**Fig. 1:**
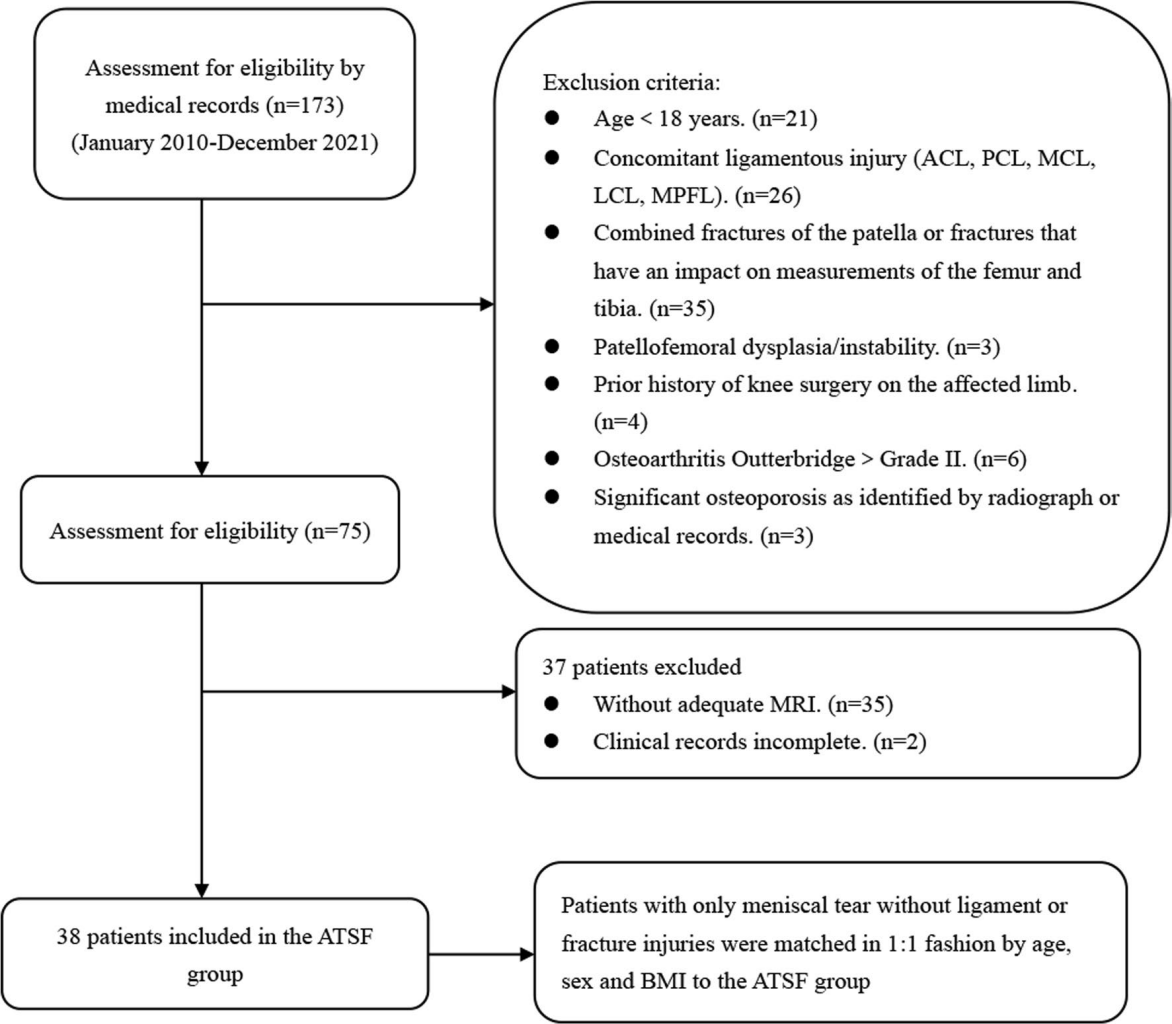
The flowchart of patient selection. *ACL* anterior cruciate ligament, *PCL* posterior cruciate ligament, *MCL* medial collateral ligament, *LCL* lateral collateral ligament, *MPFL* medial patellofemoral ligament

Before the research was started, this retrospective comparative study received institutional review board approval. Patients diagnosed with ATSF from January 2010 to December 2021 at our hospital were identified according to the hospital Electronic Medical Record System. The requirement for written informed consent was waived since this was a retrospective study, it could not cause any adverse effects for included patients, and the patient data were reported anonymously. A total of 173 patients who were diagnosed with ATSF from 2010 to 2021 were screened for eligibility by medical records (Fig. 1). Patients were excluded from the study group if they met one of the following criteria: (1) age < 18 years (because of the small number of patients), (2) concomitant ligamentous injury, (3) combined fractures of the patella or fractures that have an impact on measurements of the femur and tibia, (4) patellofemoral dysplasia/instability, (5) prior history of knee surgery on the affected limb, (6) osteoarthritis Outterbridge > Grade II, (7) significant osteoporosis. The included patients with ATSF were classified as Meyers-McKeever on radiographs or MRI [17]. During the same period, A total of 38 age-, sex- and BMI–matched patients who suffered isolated meniscus injuries were selected as the control group. Participants had any pathological condition that could affect the anatomical morphology of the knee joint, such as a discoid meniscus, were excluded from the control group. Participants with previous surgery or fractures in the lower extremity were also excluded.

### Data extraction

Patient characteristics, including age at the time of surgery, sex and BMI, were obtained from the medical records. To ensure the accuracy of the results, all the measurements were performed using good-quality MRI before surgical intervention. MRI was performed using a 1.5-T MR scanner (Magnetom Avanto, Siemens AG, Germany) with an imaging protocol composed of T1-weighted turbo spin echo (T1W-TSE) and T2-weighted fat-suppressed turbo spin echo (T2W-TSE-FS) sequences. All measurements were performed by 2 independent orthopedic surgeons using a blinded method and were repeated twice by each reviewer within an interval of 1 month to determine intraobserver and interobserver reliability. The mean of the 4 measurements was used for the corresponding final results for each patient.

**Fig. 2:**
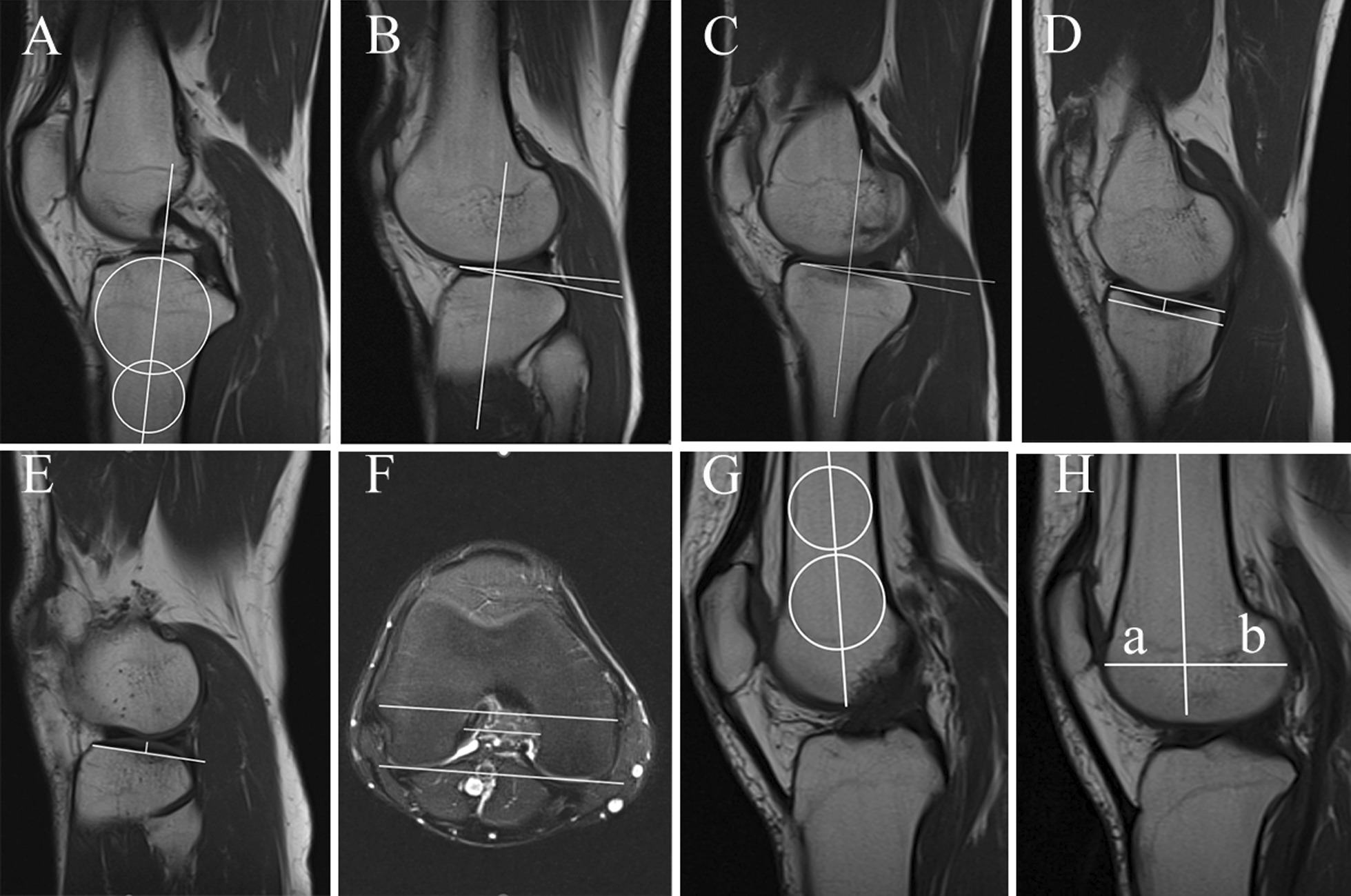
MRI images measurements performed according to the original description. **A** The anatomic axis of the tibia is determined as the line connecting the centers of 2 circles. **B** Lateral posterior tibial slope is composed of the vertical line of the anatomic axis of the tibia and the tangent to the lateral tibial plateau. **C** Medial posterior tibial slope is composed of the vertical line of the anatomic axis of the tibia and the tangent to the medial tibial plateau. **D** Medial tibial depth. **E** Lateral tibial height. **F** Notch width index. **G** The long axis of the femoral shaft. **H** Lateral femoral condyle ratio was defined as *b*/(*a* + *b*) × 100%

The lateral and medial posterior tibial slopes (LPTS and MPTS, respectively) were measured according to the method described by Hudek et al. [18]. First, to determine the anatomical axis of the tibia, the central sagittal slice of the tibia was selected, and 2 circles were placed on the proximal tibia. The proximal circle was matched with the anterior, posterior and proximal cortical borders, while the distal circle was matched with the anterior and posterior cortices. The line connecting the centers of the two circles was the anatomic axis of the tibia (Fig. 2A). The angle between the perpendicular line of the anatomical axis of the tibia and the tangent line of the lateral and medial tibial plateau is the LPTS and MPTS, respectively. This method has been reported as the most reproducible for measuring lateral tibial slope and is independent of the length of proximal tibia [19]. To determine the most predictive bony morphological risk factors, the following parameters were also measured according to the original description: medial tibial depth (MTD) [20], lateral tibial height (LTH) [21], notch width index (NWI) [22] and lateral femoral condyle ratio (LFCR) [23, 24] (Fig. 2).

### Statistical analysis

The data were checked for normal distribution by the Kolmogorov‒Smirnov test. The differences of continuous variables between the two groups were analyzed by the Student’s *t* test or Mann‒Whitney *U* test according to the normality test. Binary logistic regressions were calculated to identify the significant independent predictors of ATSF. Receiver operating characteristic (ROC) curves and the area under the curve (AUC) were constructed to evaluate the diagnostic accuracy of different parameters with the cutoffs calculated. The ideal predictive cutoff point with the highest sensitivity and specificity was determined based on the Youden index. The intraclass correlation coefficients (ICCs) were calculated to determine the intraobserver and interobserver reliability and classified as good (≥ 0.75), fair (0.50–0.74) and poor (< 0.50). Statistical analyses were performed using SPSS software (version 26; IBM). A *P* value < 0.05 was considered significant for all analyses.

## Results

A total of 38 patients who were diagnosed with ATSFs by surgery or MRI and met the inclusion criteria were included in the ATSF group. For the control cohort, 38 age-, sex- and BMI-matched participants with isolated meniscal tear were included to match the cases. The mean age of all participants was 33.64 ± 9.07 years (33.08 ± 8.53 years for the ATSF group vs. 34.21 ± 9.67 years for the control group), and the mean BMI of all participants was 23.36 ± 3.65 (22.63 ± 3.11 vs. 24.08 ± 4.04, respectively). There were no significant differences between the 2 groups in demographic characteristics (Table 1).

**Table 1:** The comparison of the demographic data between the ATSF group and control group

	ATSF (*n* = 38)	Control (*n* = 38)	*P* value
Age, year	33.08 ± 8.53	34.21 ± 9.67	0.59
Sex, M/F	15/23	15/23	1.00
BMI	22.63 ± 3.11	24.08 ± 4.04	0.083
Noncontact/contact injury	16/22	–	–
Meyers and McKeever classification			
Type I	0 (0.0)	–	–
Type II	8 (21.1)	–	–
Type III	23 (60.5)	–	–
Type IV	7 (18.4)	–	–

Values are presented as *n* (%) or mean ± SD

*ATSF* anterior tibial spine fracture, *BMI* body mass index

**Fig. 3:**
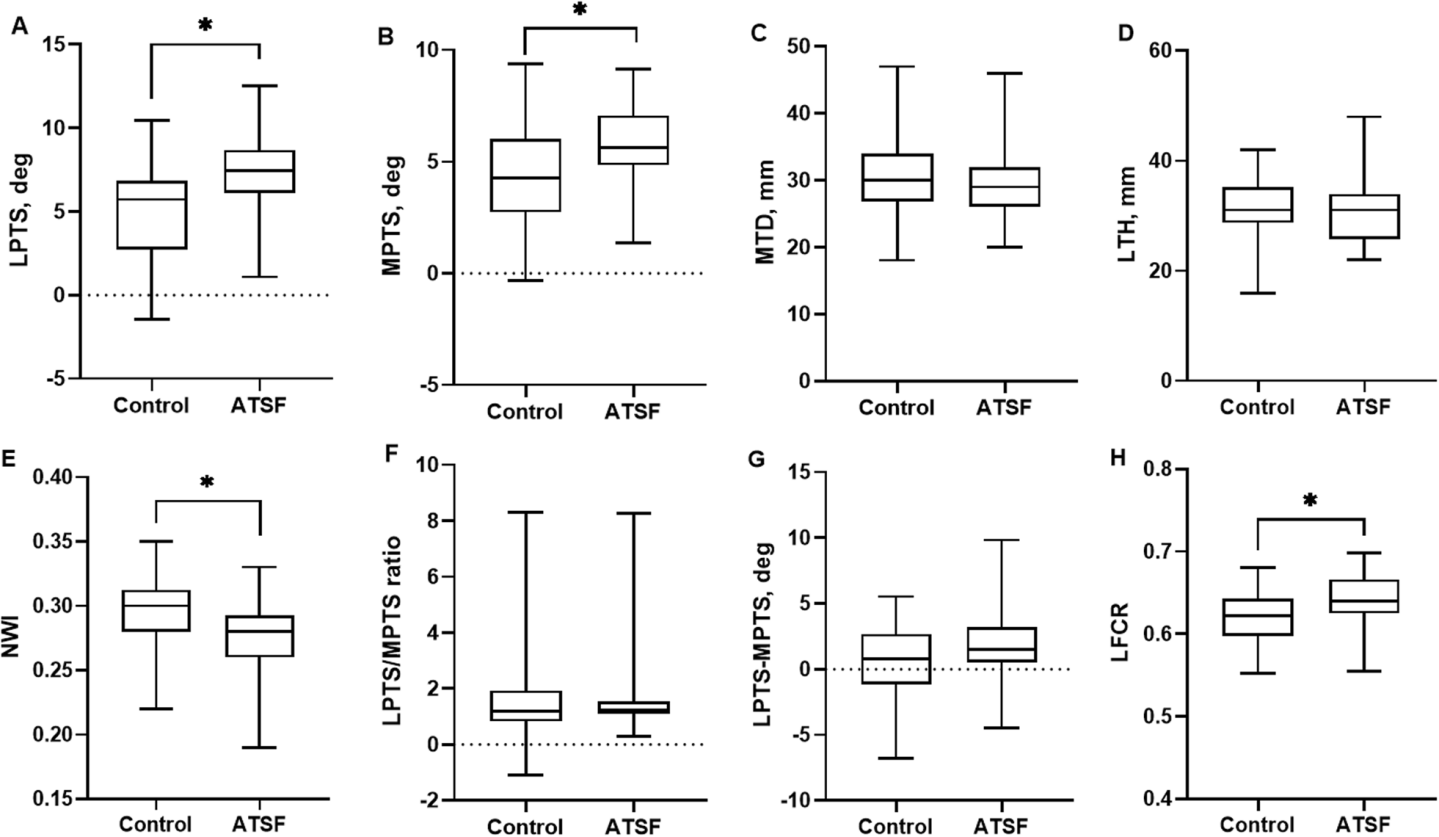
Box-and-whisker plots showed distribution of the measurement variables between the ATSF group and the control group. *ATSF* anterior tibial spine fracture. *P < 0.05. *LFCR* lateral femoral condyle ratio, *LPTS* lateral posterior tibial slope, *MPTS* medial posterior tibial slope, *MTD* medial tibial depth, *LTH* lateral tibial height, *NWI* notch width index

The MRI measurements are presented in Table 2. The intraobserver and interobserver reliabilities of measurements were categorized as good with the minimum ICCs of 0.864 and 0.851, respectively (Table 3). There were significant differences between the ATSF group and the control group regarding the following parameters: LPTS (*P* = 0.001), MPTS (*P* = 0.005), NWI (*P* = 0.005) and LFCR (*P* = 0.012). However, no significant differences were observed between groups for MTD, LTH, LPTS-MPTS and LPTS/MPTS (Fig. 3).

**Table 2:** Morphological characteristics in ATSF group and control group

	ATSF (*n* = 38)	Control (*n* = 38)	*P* value
LPTS, deg	7.42 ± 2.32	4.97 ± 3.13	**0.001**
MPTS, deg	5.78 ± 1.94	4.32 ± 2.43	**0.005**
LTS-MTS, deg	1.64 ± 2.50	0.65 ± 2.95	0.120
LTS/MTS ratio	1.52 ± 1.25	1.57 ± 1.66	0.477
LTH, mm	30.97 ± 6.42	31.32 ± 5.23	0.488
MTD, mm	29.68 ± 5.58	30.37 ± 5.87	0.411
NWI	0.28 ± 0.03	0.30 ± 0.03	**0.005**
LFCR (%)	64.05 ± 3.15	62.20 ± 3.03	**0.012**

Values are presented as mean ± SD

*ATSF* anterior tibial spine fracture, *LFCR* lateral femoral condyle ratio, *LPTS* lateral posterior tibial slope, *LTH* lateral tibial height, *MPTS* medial posterior tibial slope, *MTD* medial tibial depth, *NWI* notch width index

Bold values indicate statistical significance

**Table 3:** Inter- and intraobserver reliability expressed as ICC

	Interobserver reliability	Intraobserver reliability	
		Observer 1	Observer 2
LPTS	0.937 (0.879–0.967)	0.961 (0.937–0.976)	0.946 (0.899–0.972)
MPTS	0.924 (0.859–0.960)	0.946 (0.899–0.972)	0.931(0.872–0.964)
NWI	0.851 (0.426–0.941)	0.864 (0.629–0.941)	0.867(0.712–0.930)
LTH	0.932 (0.886–0.958)	0.929 (0.879–0.959)	0.938 (0.873–0.969)
MTD	0.872 (0.637–0.939)	0.904 (0.757–0.960)	0.942 (0.873–9.974)
LFCR	0.898 (0.859–0.929)	0.915 (0.887–0.943)	0.927 (0.895–0.955)

ICC, 2-way mixed effects model, consistency of agreement (95% confidence interval)

*ICC* intraclass correlation coefficient, *LFCR* lateral femoral condyle ratio, *LPTS* lateral posterior tibial slope, *LTH* lateral tibial height, *MPTS* medial posterior tibial slope, *MTD* medial tibial depth, *NWI* notch width index

The binary logistic regression results for the LPTS, MPTS, NWI and LFCR are shown in Table 4. From the table, the LPTS (OR = 1.326, 95% CI = 1.026–1.713, *P* = 0.031), NWI (OR = 3.1194E−14, 95% CI = 1.2831E−23–7.60E−05, *P* = 0.005) and LFCR (OR = 1.295, 95% CI = 1.063–1.578, *P* = 0.010) were found to be independently associated with ATSF.

**Table 4:** Results of multivariate logistic regression

	OR	95% CI	*P* value
LPTS	1.326	1.026–1.713	**0.031**
MPTS	1.245	0.942–1.645	0.124
NWI	3.1194E−14	1.2831E−23–7.60E−05	**0.005**
LFCR (%)	1.295	1.063–1.578	**0.010**

*LFCR* lateral femoral condyle ratio, *LPTS* lateral posterior tibial slope, *MPTS* medial posterior tibial slope, *NWI* notch width index

Bold values indicate statistical significance

**Fig. 4:**
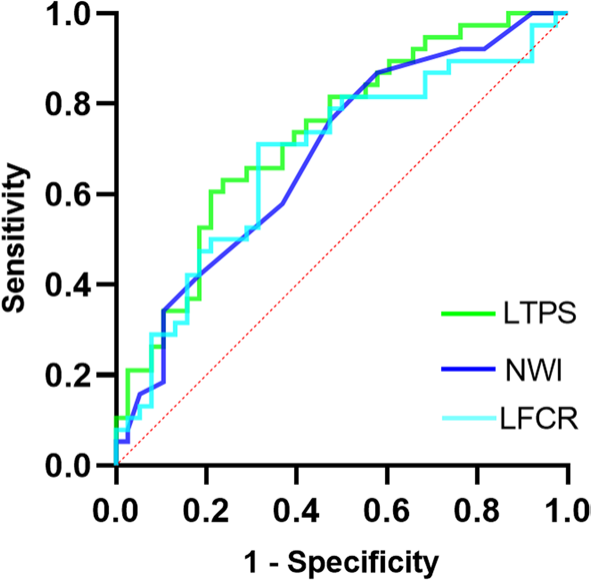
Receiver operating characteristic (ROC) analysis for significant predictor variables. *LFCR* lateral femoral condyle ratio, *LPTS* lateral posterior tibial slope, *NWI* notch width index

The ROC curves were conducted for three significant independent predictors for ATSFs to compare their diagnostic performance (Fig. 4). The most accurate predictor was the LPTS, which had a sensitivity of 63.2% and specificity of 76.3% for predicting ATSFs at an optimal cutoff of 6.9 (Table 5).

**Table 5:** Diagnostic performance of different parameters to predict ATSF

	AUC (95% CI)	*P* value	Sensitivity	Specificity	Youden index	Cutoff value
LPTS	0.731 (0.619–0.844)	< 0.001	0.632	0.763	0.395	6.9
NWI	0.686 (0.566–0.805)	0.005	0.763	0.526	0.290	0.30
LFCR (%)	0.681 (0.559–0.804)	0.007	0.711	0.684	0.395	63.04

*AUC* area under the curve, *CI* confidence interval, *ATSF* anterior tibial spine fracture, *LFCR* lateral femoral condyle ratio, *LPTS* lateral posterior tibial slope, *NWI* notch width index

## Discussion

The most important finding of this study was that LPTS, MPTS, NWI and LFCR were significantly different between patients with and those without ATSFs. However, only the LPTS, NWI and LFCR were found to be independently associated with ATSFs. The LPTS had 63.2% sensitivity and 76.3% specificity at a cutoff value of 6.9, providing the best predictive performance.

There is a paucity of research comparing the injury mechanisms of ATSFs and ACL tears. Previous investigations have shown that the demographic distribution and activity associated with ATSFs differ from those of ACL tears. Leie et al. demonstrated that skiing accounted for 56% of ATSFs, followed by soccer (22%) and rugby (16%) [25], whereas ACL tears were most prevalent in girls' soccer, followed by boys' football and basketball [26, 27]. These findings suggest that the mechanisms of these two injuries are subtly distinct. A biomechanical analysis of primate specimens has indicated that the viscoelastic properties of bone and ligaments are influenced by the rate of loading applied to the specimens [28]. Avulsion fractures are more likely to occur in cases with slower loading rates, while injuries to the intrasubstance portion of the ACL are more likely to be seen in cases with faster loading rates. In children and adolescents, discrepancies in the degree of tibial eminence ossification can also give rise to differences in injury patterns between ligament rupture and avulsion fractures.

Numerous studies have reported that increased PTS is an independent risk factor for ACL injury [13, 29, 30]. Increased PTS has an adverse effect on knee kinematics and kinetics, exerting more strain on ACL and consequently leading to stretching and tearing of ACL [31]. A recent biomechanical study found that increased PTS significantly increased tension on the ACL, and even at a 2.5° increase in PTS angle, knee joint instability and larger loading on the medial meniscus were found on the ACL-deficient knee [32]. Two studies reported the relationship between PTS and ATSF in pediatric populations, but they neither found a statistically significant association of PTS with ATSF when comparing cases with controls [11, 12]. But after stratifying by sex, Messner et al. [12] found that male patients who undergo surgical fixation of ATSF tend to have increased PTS as compared with controls. Differences with our current study in imaging modality and study populations may account for the unique findings. One of the concerns with their measurements using plain radiographs was that it was not easy to discern subtle differences between the compartments due to the superimposed medial and lateral tibial plateau, limiting their ability to analyze medial and lateral PTS, respectively [33]. Articular cartilage represents the functional point of tibiofemoral articulation, and MRI can accurately assess the 3D geometry of the cartilage surface, while plain radiographs cannot [34]. Our current study found that as the LPTS increases, for every additional degree, the risk of ATSF increases by 32.6%. And according to the results of the logistic regression analysis, MPTS was not an independent risk factor for ATSF. Although many studies have evaluated the effect of medial and lateral PTS on ACL injury, there are some conflicting findings, and LPTS measured on MRI is the most consistently reported risk factor [13, 30, 35, 36]. Our findings are in line with previous studies showing that LPTS is a more significant risk factor for ACL injury than MPTS [37–39]. Previous biomechanical literature also suggested that an increased LPTS has a greater impact on anterior tibial translation than MPTS, which creates a net internal rotation that increases the strain on the ACL [40, 41].

The association between the femoral intercondylar notch shape and dimensions and ACL injury has attracted extensive interest. In particular, NWI based on MRI measurements has widely been shown to be an important risk factor for ACL injury [42, 43]. Kocher et al. [44] evaluated skeletally immature patients with ATSF compared to matched ACL injury. They found that the ACL group had narrower notch indices than the ATSF group (0.230 vs. 0.253; *P* = 0.020). This may partially explain the different injury patterns of ATSF and ACL injury in the skeletally immature knee. Although we identified the decreased NWI as an independent risk factor for ATSF, the logistic regression results suggest that the OR value for NWI is too small to further indicate its clinical significance. The current study demonstrated that the LFCR—a novel anatomical risk factor for ACL injuries, is also correlated with ATSFs. This association may arise from the influence of the distal femoral morphology on knee kinematics. An enhanced depth of the posterior femoral condyle could induce changes in tibiofemoral interactions, ultimately leading to modifications in gait and loading mechanics, which may increase the susceptibility to ATSFs [45, 46]. Further investigations involving additional cohorts and laboratory biomechanical studies are warranted to elucidate the distinct mechanisms of ACL tears and ATSFs.

Although these anatomic risk factors are largely unmodifiable, a growing body of data highlights the role of critical anatomical parameters that can help clinicians take preventive measures to avoid injuries. Numerous studies have identified the morphological risk factors for ACL injury of the knee joint. People with a high risk of ACL injury can benefit from modifiable interventions, including landing biomechanics, neuromuscular training, balance training and improvements in playing surfaces and footwear [47]. A meta-analysis performed by Huang et al. [48] demonstrated significant protective effects of ACL injury prevention programs and reduced injury rates by 53%. Currently, although MRI is not routinely performed for screening, preoperative MRI for ATSF patients is necessary to identify other combined injuries, such as meniscus and ligament injuries, and is cost-effective. Identifying high-risk individuals through preoperative MRI allows us to individualize treatment and rehabilitation programs to improve postoperative outcomes and avoid reinjury, such as refracture and ACL injury. For individuals at high risk of ATSFs, such as skiers, it is postulated that training athletes to jump, land and cut in a biomechanical position and using appropriate athletic equipment may potentially diminish the incidence of ATSFs [9, 49]. Future research will also be warranted to determine if more conservative rehabilitation programs and return-to-sport protocols are necessary for people at high risk of ATSFs.

Limited studies have investigated morphological risk factors associated with ATSFs. To the best of our knowledge, this is the first study to confirm the relationship between ATSFs and anatomic parameters, including LPTS, MPTS, asymmetry of the medial and lateral slopes, MTD, LTH, NWI and LFCR. Our results showed that the increased LPTS and LFCR and decreased NWI are associated with an increased risk of ATSF. However, this study has certain limitations. First, due to the small number of pediatric patients, we included only adult patients, limiting the generalization of our findings to pediatric populations. Nevertheless, as the two groups exhibit different ATSF mechanisms, forthcoming studies should consider addressing them separately. Second, we screened in the hospital system based on surgical records. Patients with ATSF who did not undergo surgery were not included, especially those with Meyers and McKeever classification type I fractures. Third, we included patients with only meniscal injury in the control group. Although this method has been widely adopted by previous studies [24, 50, 51], the inclusion of this population may introduce some bias.

## Conclusion

Increased LPTS and LFCR and decreased NWI are significant risk factors for the incidence of ATSF in adults. All three parameters provide good predictive performance, but LPTS is the strongest predictor. These findings may contribute to the clinician identifying risk factors for ATSF and developing related preventive strategies. Future studies with a larger population are needed to further understand the anatomical risk factors and injury mechanisms of ATSF.

## Data Availability

All data generated or analyzed during this study are included in this article. The data are available from the corresponding author upon reasonable request.

## Abbreviations

ACL: Anterior cruciate ligament
ATSF: Anterior tibial spine fracture
PTS: Posterior tibial slope
LPTS: Lateral posterior tibial slope
MPTS: Medial posterior tibial slope
MTD: Medial tibial depth
LTH: Lateral tibial height
NWI: Notch width index
ROC: Receiver operator characteristic
